# Supplementary material for: Cardiovascular safety of celecoxib in rheumatoid arthritis and osteoarthritis patients: A systematic review and meta-analysis
Source: PLoS One. 2021 Dec 21;16(12):e0261239. doi: 10.1371/journal.pone.0261239 (PMC8691614; doi:10.1371/journal.pone.0261239)
Supplement: S1 Appendix — (DOCX) [file pone.0261239.s001.docx]

**Identification of studies via databases and registers**

Records identified from*:

MEDLINE (n=814)

CENTRAL (n=405)

EMBASE (n=1363)

CNKI (n=1440)

Wanfang (n=1429)

VIP (n =25)

Sinomed (n=803)

Total (n=6279)

Records removed *before screening*:

Duplicate records removed (n =1967)

Records marked as ineligible by automation tools (n =0)

Records removed for other reasons (n =0)

**Identification**

Records screened

(n =4312)

Records excluded**

(n =4146)

Reports sought for retrieval

(n =166)

Reports not retrieved

(n =5)

**Screening**

Reports excluded:

Not RCT (n =9)

Conference abstracts (n =4)

No needed outcomes (n =78)

No needed comparison (n=31)

Not RA/OA patient (n=4)

Same trial (n=14)

Reports assessed for eligibility

(n =161)

Studies included in review

(n =21)

Reports of included studies

(n =21)

**Included**

*Consider, if feasible to do so, reporting the number of records identified from each database or register searched (rather than the total number across all databases/registers).

**If automation tools were used, indicate how many records were excluded by a human and how many were excluded by automation tools.

*From:*  Page MJ, McKenzie JE, Bossuyt PM, Boutron I, Hoffmann TC, Mulrow CD, et al. The PRISMA 2020 statement: an updated guideline for reporting systematic reviews. BMJ 2021;372:n71. doi: 10.1136/bmj.n71

For more information, visit: <http://www.prisma-statement.org/>
