# Supplementary material for: Cardiovascular safety of celecoxib in rheumatoid arthritis and osteoarthritis patients: A systematic review and meta-analysis
Source: PLoS One. 2021 Dec 21;16(12):e0261239. doi: 10.1371/journal.pone.0261239 (PMC8691614; doi:10.1371/journal.pone.0261239)
Supplement: S3 Appendix — (PDF) [file pone.0261239.s003.pdf]

National Institute for Health Research

University of York
Centre for Reviews and Dissemination

Systematic review

*Fields that have an **asterisk (*)** next to them means that they **must be answered. Word limits** are provided for each section. You will be unable to submit the form if the word limits are exceeded for any section. Registrant means the person filling out the form.*

1. * Review title.

Give the title of the review in English

Cardiovascular Safety of Celecoxib for Rheumatoid Arthritis and Osteoarthritis: A Systematic Review and Meta-Analysis

2. Original language title.

For reviews in languages other than English, give the title in the original language. This will be displayed with the English language title.

3. * Anticipated or actual start date.

Give the date the systematic review started or is expected to start.
30/05/2020

4. * Anticipated completion date.

Give the date by which the review is expected to be completed.
30/09/2020

5. * Stage of review at time of this submission.

Tick the boxes to show which review tasks have been started and which have been completed. Update this field each time any amendments are made to a published record.

Reviews that have started data extraction (at the time of initial submission) are not eligible for inclusion in PROSPERO. If there is later evidence that incorrect status and/or completion date has been supplied, the published PROSPERO record will be marked as retracted.

This field uses answers to initial screening questions. It cannot be edited until after registration.

The review has not yet started: Yes

Review stage	Started	Completed
Preliminary searches	No	No
Piloting of the study selection process	No	No
Formal screening of search results against eligibility criteria	No	No
Data extraction	No	No
Risk of bias (quality) assessment	No	No
Data analysis	No	No

Provide any other relevant information about the stage of the review here.

6. * Named contact.

The named contact is the guarantor for the accuracy of the information in the register record. This may be any member of the review team.

Weihong Li

Email salutation (e.g. "Dr Smith" or "Joanne") for correspondence:

Dr Li

7. * Named contact email.

Give the electronic email address of the named contact.

Liweihong.403@163.com

8. Named contact address

Give the full institutional/organisational postal address for the named contact.

No. 11, Bei San Huan Dong Lu, Chaoyang District, Beijing, China

9. Named contact phone number.

Give the telephone number for the named contact, including international dialling code.

+86-15001221959

10. * Organisational affiliation of the review.

Full title of the organisational affiliations for this review and website address if available. This field may be completed as 'None' if the review is not affiliated to any organisation.

Beijing University of Chinese Medicine (BUCM)

Organisation web address:

http://www.bucm.edu.cn/

11. * Review team members and their organisational affiliations.

Give the personal details and the organisational affiliations of each member of the review team. Affiliation refers to groups or organisations to which review team members belong. **NOTE: email and country now MUST be entered for each person, unless you are amending a published record.**

Miss Bairu Cheng. Beijing University of Chinese Medicine
Miss Jiaqi Chen. Beijing University of Chinese Medicine
Mr Yuqiao Zhang. Beijing University of Chinese Medicine
Dr Weihong Li. Beijing University of Chinese Medicine
Mr Changjiang Wu. Beijing University of Chinese Medicine
Mr Qinyang Gao. Beijing University of Chinese Medicine
Mr Tenghui Li. Beijing University of Chinese Medicine
Dr Lijiao Yan. Beijing University of Chinese Medicine

12. * Funding sources/sponsors.

Details of the individuals, organizations, groups, companies or other legal entities who have funded or sponsored the review.

Funding provided by Enrollment and Employment Office of Beijing University of Chinese Medicine.

Grant number(s)

State the funder, grant or award number and the date of award

201911066

13. * Conflicts of interest.

List actual or perceived conflicts of interest (financial or academic).

None

14. Collaborators.

Give the name and affiliation of any individuals or organisations who are working on the review but who are not listed as review team members. **NOTE: email and country must be completed for each person, unless you are amending a published record.**

15. * Review question.

State the review question(s) clearly and precisely. It may be appropriate to break very broad questions down into a series of related more specific questions. Questions may be framed or refined using PI(E)COS or similar where relevant.

Is patients taking celecoxib presenting fewer, more or the same cardiovascular events than those taking non-selective NSAIDs?

16. * Searches.

State the sources that will be searched (e.g. Medline). Give the search dates, and any restrictions (e.g. language or publication date). Do NOT enter the full search strategy (it may be provided as a link or attachment below.)

Publications will be retrieved using computerized searches by EMBASE, CENTRAL, PubMed, CNKI, VIP, Wanfang and Chinese Biomedical Literature Database (CBM). No date or language limits will be set.

A re-run will be done before the final analyses.

17. URL to search strategy.

Upload a file with your search strategy, or an example of a search strategy for a specific database, (including

the keywords) in pdf or word format. In doing so you are consenting to the file being made publicly accessible. Or provide a URL or link to the strategy. Do NOT provide links to your search **results**.

Alternatively, upload your search strategy to CRD in pdf format. Please note that by doing so you are consenting to the file being made publicly accessible.

Do not make this file publicly available until the review is complete

18. * Condition or domain being studied.

Give a short description of the disease, condition or healthcare domain being studied in your systematic review.

Rheumatoid arthritis (RA) is a long-term disease characterised by persistent synovitis, systemic inflammation and autoantibodies (particularly to rheumatoid factor and citrullinated peptide). Uncontrolled active rheumatoid arthritis causes joint damage, disability, decreased quality of life, and cardiovascular and other comorbidities. But as its pathophysiology remains unclear, there is no effective treatment to it.

Osteoarthritis (OA) is a complex chronic arthritis, characterized by focal loss of articular cartilage, marginal and central new bone formation. Patients with OA often suffer from joint pain, joint swelling, knee dysfunction, and even joint deformity. Physical disability arising from pain and loss of functional capacity reduces quality of life and increases the risk of further morbidity and mortality.

Patients with both diseases often take NSAIDs to relieve pain. Since there is no evidence showing difference in cardiovascular risk between RA patients and OA patients taking NSAIDs, both patients are included in this review.

19. * Participants/population.

Specify the participants or populations being studied in the review. The preferred format includes details of both inclusion and exclusion criteria.

Inclusion: Patients with rheumatoid arthritis (diagnosed according to ACR 1987 criteria or ACR/EULAR 2010 criteria) or osteoarthritis (diagnosed according to ACR guidelines).

Exclusion: Patients with other rheumatic diseases such as systemic lupus erythematosus or Sjogren’s syndrome.

20. * Intervention(s), exposure(s).

Give full and clear descriptions or definitions of the interventions or the exposures to be reviewed. The preferred format includes details of both inclusion and exclusion criteria.

Interventions: Celecoxib at any dose.

Exclusion: Other selective NSAIDs.

NSAIDs are recommended to relieve pain for rheumatoid arthritis and osteoarthritis. They inhibit cyclooxygenase (COX), which reduces pain and inflammation through the inhibition of prostaglandins. However, the COX enzyme also presents in gastric mucosa, where it stimulates gastroprotective prostaglandins. Selective COX-2 inhibitors (such as celecoxib) offered the potential to retain efficacy while reducing gastrointestinal adverse effects. However, the cardiovascular safety of celecoxib, as compared with

non-selective NSAIDs, remains uncertain.

21. * Comparator(s)/control.

Where relevant, give details of the alternatives against which the intervention/exposure will be compared (e.g. another intervention or a non-exposed control group). The preferred format includes details of both inclusion and exclusion criteria.

Comparator: Non-selective NSAIDs or placebo.

22. * Types of study to be included.

Give details of the study designs (e.g. RCT) that are eligible for inclusion in the review. The preferred format includes both inclusion and exclusion criteria. If there are no restrictions on the types of study, this should be stated.

Only RCTs will be included.

23. Context.

Give summary details of the setting or other relevant characteristics, which help define the inclusion or exclusion criteria.

24. * Main outcome(s).

Give the pre-specified main (most important) outcomes of the review, including details of how the outcome is defined and measured and when these measurement are made, if these are part of the review inclusion criteria.

1. All cause mortality.

2. Cardiovascular mortality.

3. Fatal and non-fatal myocardial infarction.

4. Fatal and non-fatal stroke.

Measures of effect

Please specify the effect measure(s) for you main outcome(s) e.g. relative risks, odds ratios, risk difference, and/or 'number needed to treat.

For continuous variable, we will use the mean difference (MD) with 95% confidence intervals (CIs) if the measurement tool is the same. For dichotomous variable, we will use the relative risk (RR) with 95% CIs.

25. * Additional outcome(s).

List the pre-specified additional outcomes of the review, with a similar level of detail to that required for main outcomes. Where there are no additional outcomes please state ‘None’ or ‘Not applicable’ as appropriate to the review

1. Other cardiovascular events, including atrial fibrillation, arrhythmias, angina, revascularisation etc..

2. Total cholesterol (TC), triglycerides (TG), HDL, LDL.

3. Systolic blood pressure and diastolic blood pressure.

Measures of effect

Please specify the effect measure(s) for you additional outcome(s) e.g. relative risks, odds ratios, risk difference, and/or 'number needed to treat.

For continuous variable, we will use the mean difference (MD) with 95% confidence intervals (CIs) if the

measurement tool is the same. For dichotomous variable, we will use the relative risk (RR) with 95% CIs.

26. * Data extraction (selection and coding).

Describe how studies will be selected for inclusion. State what data will be extracted or obtained. State how this will be done and recorded.

Study selection:

Two reviewers will independently screen all titles and abstracts of the records. Full texts of potentially eligible studies will be retrieved for further identification according to the eligibility criteria. Any uncertainty or discrepancy will be resolved by discussion. We will use Excel for recording decisions.

Data extraction:

Two reviewers will independently extract data in accordance with a predesigned data form using Excel (version Microsoft Excel 2016). Data will be checked="checked" value="1" by an additional one reviewer. Disagreements will be resolved by discussion. Extracted information comprised the following sections:

1. General information (publication years, number of authors, the first author, study design, sample size, demographics);
2. Participants (diagnostic criteria, baseline comparison, withdrawals, loss to follow-up);
3. Interventions (dosage, administration, duration, follow-up, comparisons);
4. Outcome measures, results and adverse effects;
5. Sequence generation;
6. Allocation concealment;
7. Blinding (of participants, personnel and outcome assessors).

For missing information we will try to contact the original authors whenever possible. All discrepancies will be resolved by discussion or consulting a third reviewer.

27. * Risk of bias (quality) assessment.

State which characteristics of the studies will be assessed and/or any formal risk of bias/quality assessment tools that will be used.

Since only RCTs will be included, the risk of bias will be assessed through Cochrane's Risk-of-bias Tool for Randomized Trials (RoB 2). Two review authors will indepndently assess the risk of bias of included RCTs across six domains:

1.sequence generation;

2.allocation concealment;

3.blinding (of participants, personnel and outcome assessors);

4.incomplete outcome data;

5.selective outcome reporting;

6.other sources of bias.

For incomplete outcome data, we will report the percentage and proportion of participants lost to follow?up.

At the end, the risk of bias will be categorized as 'low', 'high' or 'unclear'. For unclear bias, we will try to contact the trial authors for more data obtaining that will could help classify as "low" or "high".

28. * Strategy for data synthesis.

Describe the methods you plan to use to synthesise data. This **must not be generic text** but should be **specific to your review** and describe how the proposed approach will be applied to your data. If meta-analysis is planned, describe the models to be used, methods to explore statistical heterogeneity, and software package to be used.

A narrative synthesis of the findings from the included studies will be provided. The GRADE method will be used to define the quality of evidence.

We will work with the data within meta-analysis, through the Review Manager 5.3. Heterogeneity related to the results of the studies will be assessed using both the ?² test and the I^2 statistic. If data are sufficiently homogenous, we will pool the results using a fixed effect model, with standardized mean differences for continuous outcomes (in our review referring to TC, TG, HDL, LDL, systolic blood pressure and diastolic blood pressure) and risk ratios for binary outcomes (in our review referring to all cause mortality, cardiovascular mortality, fatal and non-fatal myocardial infarction, fatal and non-fatal stroke, and other cardiovascular events), and calculate 95% confidence intervals and two sided P values for each outcome. We will provide summaries of intervention effects for each study by calculating risk ratios (for dichotomous outcomes) or standardized mean differences (for continuous outcomes). We will consider an I^2 value greater than 50% indicative of substantial heterogeneity. If we have high heterogeneity across included studies, we will use a random effect model or only provide a narrative synthesis of the findings from the included studies, structured around the type of intervention, target population characteristics, type of outcome and intervention content.

29. * Analysis of subgroups or subsets.

State any planned investigation of ‘subgroups’. Be clear and specific about which type of study or participant will be included in each group or covariate investigated. State the planned analytic approach.

We are not planning any subgroup analyses and although subgroup analyses may be possible or needed, it is not possible to specify the groups in advance.

30. * Type and method of review.

Select the type of review, review method and health area from the lists below.

Type of review

Cost effectiveness
No

Diagnostic

No

Epidemiologic
No

Individual patient data (IPD) meta-analysis
No

Intervention
Yes

Meta-analysis
Yes

Methodology
No

Narrative synthesis
No

Network meta-analysis
No

Pre-clinical
No

Prevention
No

Prognostic
No

Prospective meta-analysis (PMA)
No

Review of reviews
No

Service delivery
No

Synthesis of qualitative studies
No

Systematic review
Yes

Other
No

Health area of the review

Alcohol/substance misuse/abuse
No

Blood and immune system
No

Cancer
No

Cardiovascular
Yes

Care of the elderly
No

Child health
No

Complementary therapies
No

COVID-19
No

Crime and justice
No

Dental
No

Digestive system
No

Ear, nose and throat
No

Education
No

Endocrine and metabolic disorders
No

Eye disorders
No

General interest
No

Genetics
No

Health inequalities/health equity
No

Infections and infestations
No

International development
No

Mental health and behavioural conditions
No

Musculoskeletal
Yes

Neurological
No

Nursing
No

Obstetrics and gynaecology
No

Oral health

No

Palliative care
No

Perioperative care
No

Physiotherapy
No

Pregnancy and childbirth
No

Public health (including social determinants of health)
No

Rehabilitation
No

Respiratory disorders
No

Service delivery
No

Skin disorders
No

Social care
No

Surgery
No

Tropical Medicine
No

Urological
No

Wounds, injuries and accidents
No

Violence and abuse
No

31. Language.

Select each language individually to add it to the list below, use the bin icon to remove any added in error.
English

There is an English language summary.

32. * Country.

Select the country in which the review is being carried out. For multi-national collaborations select all the countries involved.
China

33. Other registration details.

Name any other organisation where the systematic review title or protocol is registered (e.g. Campbell, or The Joanna Briggs Institute) together with any unique identification number assigned by them. If extracted data will be stored and made available through a repository such as the Systematic Review Data Repository (SRDR), details and a link should be included here. If none, leave blank.

34. Reference and/or URL for published protocol.

If the protocol for this review is published provide details (authors, title and journal details, preferably in Vancouver format)

Add web link to the published protocol.

Or, upload your published protocol here in pdf format. Note that the upload will be publicly accessible.

No I do not make this file publicly available until the review is complete

Please note that the information required in the PROSPERO registration form must be completed in full even if access to a protocol is given.

35. Dissemination plans.

Do you intend to publish the review on completion?

Yes

Give brief details of plans for communicating review findings.?

We are intended to produce and submit a paper to a leading journal in this field, should the findings are considered capable of providing guidance for future trials and experiments.

36. Keywords.

Give words or phrases that best describe the review. Separate keywords with a semicolon or new line. Keywords help PROSPERO users find your review (keywords do not appear in the public record but are included in searches). Be as specific and precise as possible. Avoid acronyms and abbreviations unless these are in wide use.

37. Details of any existing review of the same topic by the same authors.

If you are registering an update of an existing review give details of the earlier versions and include a full bibliographic reference, if available.

38. * Current review status.

Update review status when the review is completed and when it is published.New registrations must be ongoing so this field is not editable for initial submission.
Please provide anticipated publication date

Review_Ongoing

39. Any additional information.

Provide any other information relevant to the registration of this review.

40. Details of final report/publication(s) or preprints if available.

Leave empty until publication details are available OR you have a link to a preprint (NOTE: this field is not editable for initial submission). List authors, title and journal details preferably in Vancouver format.

Give the link to the published review or preprint.
