# Supplementary material for: Cardiovascular safety of celecoxib in rheumatoid arthritis and osteoarthritis patients: A systematic review and meta-analysis
Source: PLoS One. 2021 Dec 21;16(12):e0261239. doi: 10.1371/journal.pone.0261239 (PMC8691614; doi:10.1371/journal.pone.0261239)
Supplement: S4 Appendix — (DOCX) [file pone.0261239.s004.docx]

**MEDLINE (via PubMed)：696 (2021.07.22: 814)**

1. arthritis, rheumatoid [mh]
2. rheumatoid OR reumatoid OR rheumatic OR reumatic OR rheumat* OR reumat* [tw]
3. arthrit* OR artrit* OR diseas* OR condition* OR nodule* [tw]
4. #2 and #3
5. felty* syndrome [tw]
6. caplan* syndrome [tw]
7. sjogren* syndrome [tw]
8. sicca syndrome [tw]
9. still* disease [tw]
10. #1 OR #4 OR #5 OR #6 OR #7 OR #8 OR #9
11. osteoarthritis [mh]
12. osteoarthr* [tw]
13. “arthritis, degenerative” OR “degenerative arthritides” OR “degenerative arthritis” OR “osteoarthrosis deformans” [tw]
14. arthros* [tw]
15. #11 OR #12 OR #13 OR #14
16. #10 OR #15
17. celecoxib [mh]
18. cyclooxygenase-2 OR cyclooxygenase-II OR “cyclooxygenase II” [tiab]
19. “cyclooxygenase 2” [tiab]
20. “cyclo oxygenase-2” OR “cyclo oxygenase 2” OR “cyclo oxygenase-II” OR “cyclo oxygenase II” [tiab]
21. cox-2 OR cox2 OR cox-II OR coxII [tiab]
22. “4-(5-(4-methylphenyl)-3-(trifluoromethyl)-1H-pyrazol-1-yl) benzenesulfonamide” [tiab]
23. celecoxib OR celebrex OR “SC 58635” OR SC-58635 OR SC58635 [tiab]
24. “cyclooxygenase inhibitors”[tiab]
25. #17 OR #18 OR #19 OR #20 OR #21 OR #22 OR #23 OR #24
26. randomized controlled trial [pt]
27. controlled clinical trial [pt]
28. randomized [tiab]
29. placebo [tiab]
30. clinical trials as topic [mesh:noexp]
31. randomly [tiab]
32. trial [ti]
33. #26 OR #27 OR #28 OR #29 OR #30 OR #31 OR #32
34. animals [mh] NOT humans [mh]
35. #33 NOT #34

#36 #16 AND #25 AND #35

**Cochrane CENTRAL：Trials=212 Trials=405(2021.07.22)**

1. (cyclooxygenase-2 or cyclooxygenase 2 or cyclooxygenase-II or cyclooxygenaseII).ti,ab.

2. (cyclo oxygenase-2 or cyclo oxygenase 2 or cyclo oxygenase-II or cyclo oxygenaseII).ti,ab.

3. (cox-2 or cox2 or cox-II or coxII).ti,ab.

4. (celecoxib or celebrex or SC-58635).ti,ab.

5. MeSH descriptor: [Cyclooxygenase Inhibitors] explode all trees

6. or/1-5

7. MeSH descriptor: [Osteoarthritis] explode all trees

8. (arthrit$ or osteoarthrit$).ti,ab,kw

9. or/7-8

10. MeSH descriptor: [Arthritis, Rheumatoid] explode all trees

11. ((rheumatoid or reumatoid or revmatoid or rheumatic or reumatic or revmatic or rheumat$ or reumat$ or revmarthrit$) adj3 (arthrit$ or artrit$ or diseas$ or condition$ or nodule$)).tw.

12. (felty$ adj2 syndrome).tw.

13. (caplan$ adj2 syndrome).tw.

14. (sjogren$ adj2 syndrome).tw.

15. (sicca adj2 syndrome).tw.

16. still$ disease.tw.

17. or/10-16

18. 6 and (9 or 17)

**EMBASE(via Ovid):（1290）（2021.07.22: 1363）**

1. (cyclooxygenase-2 or cyclooxygenase2 or cyclooxygenase-II or cyclooxygenaseII).ti,ab.

2. (cyclo oxygenase-2 or cyclo oxygenase2 or cyclo oxygenase-II or cyclo oxygenaseII).ti,ab.

3. (cox-2 or cox2 or cox-II or coxII).ti,ab.

4. (celecoxib or celebrex or SC-58635).af.

5. Cyclooxygenase 2 inhibitor/

6. Cyclooxygenase 2/

7. Celecoxib/

8. or/1-7

9. arthritis, rheumatoid/

10. (felty$ adj2 syndrome).tw.

11. (caplan$ adj2 syndrome).tw.

12. rheumatoid nodule.tw.

13. (sjogren$ adj2 syndrome).tw.

14. (sicca adj2 syndrome).tw.

15. still$ disease.tw.

16. bechterew$ disease.tw.

17. (arthritis adj2 rheumat$).tw.

18. or/9-17

19. osteoarthritis/

20. osteoarthr$.tw.

21. (degenerative adj2 arthritis).tw.

22. or/19-21

23. random$.tw.

24. factorial$.tw.

25. placebo$.tw.

26. (doubl$ adj blind$).tw.

27. (singl$ adj blind$).tw.

28. assign$.tw.

29. allocat$.tw.

30. randomized controlled trial/

31. randomization/

32. Randomi?ed controlled trial$.tw.

33. Rct.tw.

34. random allocation.tw.

35. allocated randomly.tw.

36. (allocated adj2 random).tw.

37. or/23-36

38. 18 or 22

39. 8 and 37 and 38

中国知网：1189 (2021.07.22: 1440)

TKA=('塞来昔布'+'COX-2'+'西乐葆'+'环氧酶化酶-2'+'SC 58635'+'SC-58635') AND TKA=('类风湿'+'关节炎'+'骨关节病') AND (TKA=('随机'+'试验') NOT TI='鼠')

万方：1092 (2021.07.22: 1429)

(主题:("塞来昔布" OR "COX-2" OR "西乐葆" OR "环氧酶化酶-2" OR "SC-58635" OR"SC 58635") AND ("类风湿" OR "关节炎" OR "关节病")) AND (主题: ("随机" OR "试验") NOT 题名:"鼠")

主题词扩展

重庆维普：24 (2021.07.22: 25)

(U=(塞来昔布 OR “SC 58635” OR SC-58635 OR COX-2 OR 环氧酶化酶-2) AND (类风湿 OR 关节炎 OR 骨关节病)) AND (U=(随机 OR 试验) NOT T=鼠)

Sinomed：681 (2021.07.22: 803)

(("随机对照试验"[常用字段:智能] OR "随机"[常用字段:智能] OR "试验"[常用字段:智能]) NOT "鼠"[标题]) AND ("塞来昔布"[常用字段:智能] OR "cox-2"[常用字段:智能] OR "环氧酶化酶-2"[常用字段:智能]) AND ("类风湿"[常用字段:智能] OR "骨关节炎"[常用字段:智能])
