# Supplementary material for: Cardiovascular safety of celecoxib in rheumatoid arthritis and osteoarthritis patients: A systematic review and meta-analysis
Source: PLoS One. 2021 Dec 21;16(12):e0261239. doi: 10.1371/journal.pone.0261239 (PMC8691614; doi:10.1371/journal.pone.0261239)
Supplement: Attachment — Submitted filename: Response to Reviewers.docx [file pone.0261239.s008.docx]

Reviewer #1: This is a timely manuscript which aims to provide some higher level of evidence in a relative arguable subject, namely the overall and in particular the cardiovascular safety profile of celecoxib and NSAIDs in patients with OA and/or RA. The authors performed a systematic review and a meta-analysis of available literature and found that celecoxib has a better CV profile than other NSAIDs in terms of all-cause mortality and CV mortality, whereas no differences have been observed for myocardial infarction and CVA. Similarly, no differences have been shown with respect to all analyzed outcomes when celecoxib has been compared to placebo. Some points could be made that should be addressed in order to improve the manuscript:
1. English language: please have the manuscript checked by a native English speaker or someone with highest qualifications in English language.

Response: We apologize for the insufficient quality of the original manuscript. This issue has been highly recognized by our team, and multiple times of revisions with great scrutiny have been performed. Some grammatical errors and expressions have been checked. We hope we have presented a better version. Thank you.

2. Methodologic issues/statistical issues: the study of Nissen (2016) and MacDonald (2017) are the largest studies and both of them provide data supporting a neutral/beneficial effect of celecoxib with respect to all-cause mortality and CV mortality. Likewise, it is of little surprise that the meta-analysis imbedding this two studies would come to the same conclusion, only now reaching statistical significance. My point is that we should still be caution when interpreting the results, because even this is a meta-analysis, it seems that these two studies are actually the ones determining the final outcome. Could the authors discuss this point?

Response: Thank you for your question. If the amount of included literature meta-analysis is small, such as two studies as you mentioned, there is often a serious bias. In principle, we can see whether there is publication bias through the funnel chart for more than 9 documents. Publication bias is an important aspect which should be considered because in many cases, articles with negative results are less likely to be published. As for other biases, we can only find out based on the evaluation of the quality of the literature (criteria and methodology). The available literature should be included in the study according to the PRISMA guidelines.

3. Clinical relevance: from the clinician point of view it is of relevance to know if it is possible to safely prescribe NSAIDs to a patient, and which one fits the best with his clinical profile. Among the studies included in this meta-analysis a few investigated RA patients. Is it possible to perform separate analysis for the OA and RA patients respectively? While NSAIDs are often prescribed as painkillers in OA (where paracetamol represents an equally good alternative, given the low-inflammatory status in OA), their prescription in RA can sometimes be determined by an increasing of disease activity, thus working more as anti-inflammatory drugs, decreasing inflammation and consequently its detrimental cardiovascular effects. Therefore one may hypothesize that the same drug (celecoxib) might have different CV impact in RA and OA respectively because of different biological effects (more painkilling (OA) vs. more anti-inflammatory (RA)).
It would therefore be very useful if the authors would provide the readers with an answer to the following questions (based on their results):
a) Can I safely prescribe celecoxib in an OA patient? As a first NSAID? What if the patient has a history of CV events?
b) Can I safely prescribe celecoxib to an RA patient? As a first NSAID? What if the patient has a history of CV events?
Thank you.

Response: Thank you for your question. Actually, before the submission, we tried to do this subgroup analysis you mentioned but failed because only 1 of the 24 trials included in our systematic review recruited only RA patients. 3 trials recruited both OA and RA patients, but data of OA and RA subgroups were not provided. Therefore, it is difficult to do this subgroup analysis. But based on the evidence we have so far, we consider it safe to prescribe celecoxib at an approved dose - 100, 200, and 400mg per day - in an OA patient, as a recommended NSAID. But we dare not say “a first NSAID” because naproxen also showed great safety and no statistical differences have been found between them in the study of Essex (2012)[1], MacDonald (2017)[2], Nissen (2016) [3] and Simon (1999)[4]. As for RA patients, although the participants were fewer, we still think it’s OK to draw the same conclusion because 1) any enhancement of CV risk with celecoxib or nsNSAID is modest[2]; and 2) trials with OA&RA patients presented the same results as those of trials with only OA patients. However, no subgroup analysis was reported considering patients with/without a history of CV events, so we cannot give a sound conclusion so far. We have added some related contents in the CONCLUSION.

Reviewer #2: Liu et al. have written a systematic review and meta-analysis about the use of celecoxib and its cardiovascular safety in patients with rheumatoid arthritis and osteoarthritis. This is a relevant topic for daily clinical practice. However, I have several concerns about the manuscript.

1. In the introduction, on page 2, line 47 and 48, the authors write about coronary atherosclerosis and non-fatal myocardial infarction. I think this sentence should be refrased. Now it reads as if coronary atherosclerosis and a myocardial infarction are different entities. It could be refrased to "particularly (fatal and non-fatal) myocardial infarction due to coronary atherosclerosis".

Response: Thank you for your advice and we apologize for the insufficient quality of the original manuscript. We value your opinion very much and have rephrased it as you suggested.

2. The authors write that there are no specific treatment strategies for CV disease in RA patients. However, the EULAR recommendations state that there is evidence (from mainly observational studies) that an optimal control of disease activity and treatment of CVD risk factors reduces the CVD risk in patients with an inflammatory joint disease.

Response: Thank you for your comments. Our purpose by saying “there were no specific treatment strategies” was that the relationship between RA and its raised CV risk remains unclear. Therefore, no specific treatments could have been found. We deeply agree with the EULAR recommendations that controlling disease activity and treatment of the risk factors reduces the CVD risk. To minimize misunderstanding, we deleted related lines.

3. Regarding the statistical analysis I would like to advise to discuss the methods with a statistician. Studies with zero events were included in the analyses. I wonder if this is the correct way to do these analyses. I wonder if you can conclude that all-cause mortality was decreased in the celecoxib group when there are 0-2 events in the studies included.

Response: Thanks for the advice. We discussed the methods with a statistician (Jingli Xing), and she found no mistakes in them. In a meta-analysis, when we want to calculate the rate of a specific event, the exact number of the event should be recorded, even though sometimes this event did not happen - meaning the rate is 0%. In Cochrane Handbook for Systematic Reviews of Interventions Version 6.2, Chapter 10.4.4.2: Studies with no events in either arm, it wrote: “Whilst the results of risk difference meta-analyses will be affected by non-reporting of outcomes with no events, odds and risk ratio based methods naturally exclude these data whether or not they are published, and are therefore unaffected”[5]. Therefore, we think it is appropriate to include studies with zero events.

4. I could not find which non-selective NSAIDs the authors included in their search and comparison with celecoxib. I think this is relevant and should be added to the manuscript.

Response: Thanks for your question. The details about which non-selective NSAIDs, if any, were included in each trial are concluded in “Table 1 Characteristics of randomized controlled trials included in qualitative synthesis”, which contains ibuprofen, diclofenac, naproxen, and dexiprofen. In the PROBE trial, the nsNSAID in the comparator group was any nsNSAID of the investigator’s choice, prescribed within the dosages allowed in the United States package insert. In most of the trials, celecoxib was compared with placebo. As the incidence of events decreases, the logarithmic conversion, logit conversion, and arcsine conversion methods can still maintain good performance.

5. In general, I would advise to ask a native English speaker to review the manuscript.

Response: We apologize for the insufficient quality of the original manuscript. This issue has been highly recognized by our team, and multiple times of revisions with great scrutiny have been performed this time. Some grammatical errors and expressions have been checked. We hope we have presented a better manuscript. Thank you.

References:

[1]	ESSEX M N, BHADRA P, SANDS G H. Efficacy and tolerability of celecoxib versus naproxen in patients with osteoarthritis of the knee: a randomized, double-blind, double-dummy trial [J]. J Int Med Res, 2012, 40(4): 1357-70.

[2]	MACDONALD T M, HAWKEY C J, FORD I, et al. Randomized trial of switching from prescribed non-selective non-steroidal anti-inflammatory drugs to prescribed celecoxib: the Standard care vs. Celecoxib Outcome Trial (SCOT) [J]. Eur Heart J, 2017, 38(23): 1843-50.

[3]	NISSEN S E, YEOMANS N D, SOLOMON D H, et al. Cardiovascular Safety of Celecoxib, Naproxen, or Ibuprofen for Arthritis [J]. N Engl J Med, 2016, 375(26): 2519-29.

[4]	SIMON L S, WEAVER A L, GRAHAM D Y, et al. Anti-inflammatory and upper gastrointestinal effects of celecoxib in rheumatoid arthritis: a randomized controlled trial [J]. JAMA, 1999, 282(20): 1921‐8.

[5] Deeks JJ, Higgins JPT, Altman DG (editors). Chapter 10: Analysing data and undertaking meta-analyses. In: Higgins JPT, Thomas J, Chandler J, Cumpston M, Li T, Page MJ, Welch VA (editors). Cochrane Handbook for Systematic Reviews of Interventions version 6.2 (updated February 2021). Cochrane, 2021. Available from www.training.cochrane.org/handbook.
