# Supplementary material for: Cardiovascular safety of celecoxib in rheumatoid arthritis and osteoarthritis patients: A systematic review and meta-analysis
Source: PLoS One. 2021 Dec 21;16(12):e0261239. doi: 10.1371/journal.pone.0261239 (PMC8691614; doi:10.1371/journal.pone.0261239)
Supplement: Attachment — Submitted filename: Response to Reviewers.docx [file pone.0261239.s009.docx]

Reviewer #1:

Q1: I would like to ask the authors to address in the Discussion the issue of publication bias, as an important limitation to their meta-analysis.

Response: Thanks for your advice. As the number of trials included in each meta-analysis was small (fewer than 10 trials), publication bias could not have been assessed properly, which makes it an important limitation to our work. We have addressed this issue in the Discussion (Line 275-276).

Q2: Secondly, I would like to know if there is an explanation for the fact that 11 studies mentioned in Table 1 (references no. 25,26, 28, 29, 32, 34, 36, 38, 41, 42, 45) can not be found among the studies presented in the figures 4 - 8. Is the data from these 11 studies excluded from the meta-analysis?
Response: Thanks for your question. Figures 4-8 presented part of the pooled results of the primary outcomes, which are all-cause mortality, cardiovascular mortality, myocardial infarction, and stroke. Some negative results were only shown in the text because we were trying to reduce the number of pictures. As for the secondary outcomes, which are atrial fibrillation, arrhythmias, angina, revascularization, and heart failure, there were no figures in our last edition of the manuscript because some were only reported in single studies, some in no studies, and none of the results was statistically significant. We value your opinion very much and agree with you that it’s improper that we did not give more details about the 11 studies you mentioned, so we combined all the results of dichotomous outcomes into 2 figures (figure 4 and 5 in our latest manuscript), where 19 of the 21 studies included can be found except Sampalis 2012^[1]^ and Simon 1999^[2]^ for following reasons: 1. Sampalis 2012 did not present any data capable for analysis, so we only described their results in the text; 2. Simon 1999 was the only study that presented blood pressures in the celecoxib group and placebo group before and after the study, so the results couldn’t be pooled and also couldn’t be shown in **Fig 5. Relative risk of dichotomous outcomes for celecoxib versus placebo** as they were continuous data. Therefore, we only described their findings in the text.

Q3: Finally, I would like to ask the authors to sharpen their aim of the study at the end of the introduction and also to address in their discussion or conclusion the gain of knowledge for the scientific community resulting from their meta-analysis. Thank you.

Response: Thanks for your advice. We also think doing so helps readers to better find their take-home messages, so some changes were made in the text (Line 84-86, and Line294-295) to address the aim and findings clearer.

Thank you again for all your precious comments! They’ve been really helpful to us and this article.

**References:**

[1]	SAMPALIS J S, BROWNELL L A. A randomized, double blind, placebo and active comparator controlled pilot study of UP446, a novel dual pathway inhibitor anti-inflammatory agent of botanical origin [J]. Nutr J, 2012, 11(21.

[2]	SIMON L S, WEAVER A L, GRAHAM D Y, et al. Anti-inflammatory and upper gastrointestinal effects of celecoxib in rheumatoid arthritis: a randomized controlled trial [J]. JAMA, 1999, 282(20): 1921‐8.
